# Subcutaneous fat thickness, but not epicardial fat thickness, parallel weight reduction after bariatric surgery: a cardiac magnetic resonance study

**DOI:** 10.1186/1532-429X-15-S1-E54

**Published:** 2013-01-30

**Authors:** Murilo Foppa, Kyle Pond, Daniel D Jones, Kraig V Kissinger, Beth Goddu, Benjamin Schneider, Rahul Jhaveri, Warren J Manning

**Affiliations:** 1 Cardiology, Hospital de Clinicas de Porto Alegre, Porto Alegre, Brazil; 2 Medicine (Cardiovascular Division), Beth Israel Deaconess Medical Center/Harvard Medical School, Boston, MA, USA; 3 Radiology, Beth Israel Deaconess Medical Center/Harvard Medical School, Boston, MA, USA; 4 Surgery, Beth Israel Deaconess Medical Center/Harvard Medical School, Boston, MA, USA

## Background

Bariatric surgery is a very effective treatment for morbid obesity, generally improving the obesity related metabolic derangements. Epicardial fat (EPIFAT) is a visceral fat depot measurable in cardiac magnetic resonance. Changes in EPIFAT after bariatric surgery has been described, but it is not well characterized. We hypothesized that the reduction of the thoracic fat components after bariatric surgery is heterogeneous.

## Methods

We analyzed since steady state free precession (SSFP) CMR data from 13 patients (51±11years; BMI=44±4 kg/m2; XX F) before and 90±13 days after bariatric surgery (BS). Data were compared with 11 control, subjects (65±7 years; BMI=27.2 kg/m2; XX F) who had two CMR exams 55±30 days apart (CTR) but not submitted to an obesity intervention. From cine SSFP images we measured: 1) EPIFAT: mean of the maximal visceral fat thickness anterior to the epicardial surface on the right ventricular (RV) free-wall on the HLA and 4 chamber views, and the maximal fat thickness anterior to the interventricular groove on the basal short axis view, 2) Paracardial fat (PARAFAT): the fat thicknesses over the RV free-wall outside the pericardium on the HLA and 4 Chamber views, and 3) Subcutaneous fat (SUBFAT): the maximal subcutaneous fat thickness measured at the mid sternal level.

## Results

Baseline and follow-up SUBFAT, PARAFAT and EPIFAT measurements for BS and CTR groups are displayed in Table 1. At follow-up, the BS group weight declined by 18±3.9 kg (16±3.7% of body mass). There was no body weight change in the CTR group. In the BS group, the corresponding change in the fat components were SUBFAT= -10.1±4.7 mm (P<0.001), PARAFAT= -1.2±3.1 mm (P=0.2), and EPIFAT= -0.5±1.3 mm (P=0.2). The correlation coefficients between the fat components reduction and weight loss were SUBFAT: r=0.63 (P=0.03), PARAFAT: r=0.64 (P=0.02), and EPIFAT: r=0.22 (P=0.5). In the CTR group, we tested the reproducibility between the two CMR studies. The intraclass correlation coefficients were higher for SUBFAT=0.93 and PARAFAT=0.94, but lower for EPIFAT=0.35.

**Table 1:** Fat thickeness (mm) in bariatric surgery and control groups

	Baseline	Follow-up	P-value
SUBFAT			

Bariatric	33.3±9.2	23.2±8.4	<0.001

Control	16.5± 4.5	15.3± 4.2	0.1

PARAFAT			

Bariatric	11.8±2.9	10.5±1.6	0.2

Control	9.5±3.5	9.4±3.4	0.4

EPIFAT			

Bariatric	5.8 ±1.7	6.3±1.4	0.2

Control	6.3±0.9	6.2±0.7	0.7

## Conclusions

Three months after bariatric surgery, there is a significant reduction in SUBFAT, which parallels the reduction in body weight. However, there is no significant reduction in the PARAFAT thickness. Additionally, we could not demonstrate significant EPIFAT reduction or relationship with weight loss.

## Funding

CAPES (Brazilian Research Agency) n 9601/11.

